# Application of remimazolam-0.6% sevoflurane anesthesia for flash visual evoked potential monitoring during pituitary adenoma resection: a non-inferiority randomized controlled trial

**DOI:** 10.1186/s12871-024-02466-0

**Published:** 2024-02-29

**Authors:** Fu Shi, Ranran Tang, Xiangrong Du, Xin Li, Guisheng Wu

**Affiliations:** 1 Department of Anesthesiology, Liaocheng People’s Hospital, No. 67. Dongchang West Road, Liaocheng, Shandong 252000 China; 2 Department of Neurosurgery, Liaocheng Brain Hospital affiliated Liaocheng People’s Hospital, No. 45. Huashan Road, Liaocheng, Shandong 252000 China

**Keywords:** Flash visual evoked potential, Remimazolam, Propofol, Pituitary adenoma resection, Electroretinogram

## Abstract

**Background:**

Flash visual evoked potential (FVEP) is a critical method for monitoring intraoperative visual function during neurosurgery. A new benzodiazepine drug called remimazolam has recently been used for general anesthesia. However, the impact of remimazolam on FVEP remains unclear. Therefore, we aimed to investigate how remimazolam, in comparison to propofol, when combined with 0.6% sevoflurane anesthesia, affects the FVEP waveform during pituitary adenoma resection.

**Methods:**

Overall, 36 patients undergoing pituitary adenoma resection under general anesthesia were randomly assigned to either the remimazolam group (Group R) or the propofol group (Group P) in a prospective, randomized, controlled, non-inferiority trial. For anesthesia induction, a bolus of 0.2 mg/kg remimazolam or 2 mg/kg propofol was intravenously infused for approximately one minute. The anesthesia was maintained by continuous infusion of either remimazolam (0.7-1.0 mg/kg/h) or propofol (4–6 mg/kg/h), in combination with 0.6% sevoflurane, aimed at sustaining the bispectral index (BIS) within the range of 40–60. The primary outcome was the N75-P100 amplitude of FVEP recorded at approximately 20 min after intubation (T0). 10% of the amplitude at T0 in group P was defined as the non-inferiority margin (δ). Confidence interval testing was used to evaluate the non-inferiority hypothesis. The secondary outcomes covered the P100 latency of FVEP, electroretinogram (ERG) b wave amplitude, demographic characteristics, hemodynamics, and occurrence of adverse events.

**Results:**

The BIS index during anesthesia was comparable between the groups at the same measured time points (*P* > 0.05). The N75-P100 amplitude at T0 in group R was 7.64 ± 1.36 µV, while it was 6.96 ± 0.95 µV in group P (*P* = 0.09), with a mean difference of 0.68 µV (95% CI, -0.11 µV to 1.48 µV). The δ was set at 0.7 and the lower limit of the 95% CI exceeded the -δ. Both remimazolam and propofol had little effect on ERG b-wave amplitudes. At the designated time points, FVEP amplitude and P100 latency displayed no appreciable variation between the two groups (*P* > 0.05). Furthermore, there were no significant differences in the incidence of adverse events related to anesthesia, needle electrodes, or surgery between the two groups (*P* > 0.05).

**Conclusion:**

Our findings suggest that remimazolam-0.6% sevoflurane is non-inferior to propofol-0.6% sevoflurane for general anesthesia, based on the FVEP N75-P100 amplitude. The electrophysiological data obtained in both groups indicate that reproducible and stable FVEP and ERG waveforms can be acquired at set time points. Therefore, for reliable FVEP monitoring, remimazolam-0.6% sevoflurane appears to be a safe and effective protocol in general anesthesia.

**Trials registration:**

This study was registered on chictr.org.cn (ChiCTR2200056803, 17/02/2022).

## Introduction

Pituitary adenomas are commonly benign with an approximate prevalence of 1/1000, and endoscopic transsphenoidal resection is the preferred surgical method for pituitary adenoma [1, 2]. Because of the anatomic location of pituitary adenoma is close to the optic chiasm, neurosurgical procedures may carry a potential risk of visual function deterioration. Intraoperative visual pathway impairment will seriously increase the concerns in patients with preserved visual function. During tumor resection, monitoring flash visual evoked potentials (FVEP) can assess the functional integrity of the optic pathway from the retina to the visual cortex [3]. By observing the amplitude and latency changes of FVEP, the surgeon can be guided to choose the surgical path to effectively avoid or reduce the occurrence of postoperative visual function damage.

Early in 2010, Sasaki, T et al. reported that the waveform changes of intraoperative FVEP and the prognosis of visual function had a significant correlation, confirming the application value of intraoperative FVEP monitoring [4]. Similarly, Nishimura, F et al. further verified the satisfactory effectiveness and sensitivity of FVEP in predicting postoperative visual function [5]. However, the FVEP waveform is prone to inhibition by both common anesthetics and physiological factors owing to the multisynaptic nature of the visual conduction pathway. Thus, seeking a proper anesthesia strategy that can acquire a reliable quality of FVEP during surgery is a challenging task for anesthesiologists.

Neurophysiological monitoring is easily affected by inhaled halogenated anesthetics. A previous study has revealed the FVEP could not be interpreted and recordable when sevoflurane concentrations reach 1.71% [6]. Compared with sevoflurane, propofol-based general anesthesia has generally been performed in neurosurgery when monitoring FVEP. Hayashi and Kawaguchi [7] showed that propofol had a relatively small suppressive effect on FVEP. Furthermore, it was demonstrated that all the FVEP waveforms of 19 patients can be elicited under total propofol intravenous anesthesia, with high VEP amplitude and short latency. However, propofol also has its own drawbacks. FVEP amplitude may also be suppressed by propofol when administered in a large dose (4 µg/ml) [8]. In some muscle relaxants free situations, high dose of propofol tends to cause considerable hemodynamic fluctuations. It is particular need to explore new options for suitable anesthesia regimens to facilitate measurement and tracking of FVEP. Coincidentally, a recent study chosen the balance anesthesia regimen for FVEP monitoring and demonstrated the propofol combined with a low concentration of sevoflurane was non-inferior to total propofol intravenous anesthesia [9].

In recent years, remimazolam tosilate, developed by Jiangsu Hengrui Pharmaceutical Co., Ltd. in China, has been confirmed to have sedative effects on γ-aminobutyrate subtype A (GABA_A_) receptors [10], and we have used it for general anesthesia in a previous study [11]. To data, very few case reports have explored the impact of remimazolam on neurological evoked potential monitoring, such as motor, visual, and sensory evoked potentials during neurosurgery [12–14]. Although the results suggested that remimazolam may be a candidate to propofol for evoked potential monitoring, the existing data are insufficient to explain whether remimazolam is beneficial to FVEP monitoring. Based on our clinical experience, balanced anesthesia can provide adequate depth of anesthesia, hemodynamic stability, and safety. Therefore, we try to explore the effect of remimazolam and propofol combined with 0.6% sevoflurane on the waveform of FVEP.

Our goal was to test the effect of remimazolam-0.6% sevoflurane anesthesia on the waveform of FVEP is non-inferior to that of propofol-0.6% sevoflurane anesthesia. To verify our hypothesis, we conducted a prospective, randomized, controlled and non-inferiority trial in adult patients who underwent pituitary tumor resection requiring FVEP monitoring.

## Materials and methods

### Study design

This was a prospective, single-center, randomized, controlled, non-inferiority trial. The Ethics Committee (Liaocheng People’s Hospital in Shandong Province, China) approved this study (No. 2,021,024). After signing the informed consent form, the patients were qualified to participate in the study. This protocol was also registered at the Chinese Clinical Trial Registry on February 17, 2022 (ChiCTR2200056803, main researcher: Fu Shi). Patients undergoing pituitary adenoma resection under general anesthesia that required intraoperative flash visual evoked potential (FVEP) monitoring were consecutively included in this study from March 2022 to May 2023. The patients with pituitary adenoma were confirmed by pathology after tumor resection and were aged from 19 to 77 years old, with American Society of Anesthesiologists (ASA) physical status I to III. We excluded patients with severe visual dysfunction (corrected visual acuity < 0.4), uncontrolled hypertension, diabetes and arrhythmia, unable to elicit FVEP after anesthesia induction, severe liver and kidney insufficiency, allergy to drugs used in this study, a history of ocular illness (cataract and glaucoma) or ophthalmic surgery, mental diseases or inability to communicate, and refusal to sign the informed consent form.

### Randomization and blinding

A random number list was generated at a 1:1 ratio using a computer. As patients continued to join, the subjects were equally assigned to two groups by the random sequence number (n = 18 patients in each group). An anesthesiologist prepared the anesthetics and performed the preset anesthesia protocol according to group allocation. The FVEP data recording and analysis were implemented by a trained neurophysiology technician who was blind to the anesthesia design. All surgical procedures were performed by the same experienced neurosurgeon. The outcome collection and evaluation were performed by another anesthesiologist. All participants were blinded to group assignment throughout the study.

### Anesthesia protocol

Preoperative drugs, such as phenobarbital sodium or atropine, were not administered to all patients. After being transferred to the theater, standard vital sign monitoring, such as electrocardiogram (ECG), heart rate (HR), invasive blood pressure (IBP), pulse oxygen saturation (SpO_2_), end-tidal carbon dioxide partial pressure (PetCO_2_) and bispectral index (BIS) was performed for all patients.

In the group P, propofol (2.0 mg/kg), cisatracurium (0.2–0.25 mg/kg) and sufentanil citrate (0.3 µg/kg) were given for anesthesia induction, and propofol (4.0–6.0 mg/kg/h) and remifentanil (0.1–0.3 µg/kg/h) were infused for anesthesia maintenance. For the patients in the group R, the anesthesiologist used remimazolam (0.2 mg/kg), cisatracurium (0.2–0.25 mg/kg) and sufentanil citrate (0.3 µg/kg) for anesthesia induction and administered remimazolam (0.7-1.0 mg/kg/h) and remifentanil (0.1–0.3 µg/kg/h) for anesthesia maintenance. After an endotracheal tube was inserted, sevoflurane (concentration: 0.6%, mixed with 2 L/min oxygen) was inhaled for the two groups. Thereafter, the BIS was maintained at 40–60 by adjusting the infusion rate of propofol or remimazolam. Mechanical ventilation parameters, such as tidal volume and respiratory frequency were adjusted to keep the PetCO_2_ at 30–40 mmHg. In our study, mean arterial blood pressure (MAP) less than 65 mmHg was regarded as hypotension and HR < 55 bpm was defined as bradycardia. Atropine (0.4 mg) and ephedrine (0.1 mg/kg, IV) were intravenously administered to keep the blood circulation stable. After surgery, all patients were routinely transferred to the postanesthesia care unit (PACU) to discover adverse events related to FVEP monitoring and general anesthesia in a timely manner.

### FVEP and ERG monitoring method

After intubation, a total of 7 subcutaneous needle electrodes (Friendship Medical Electronics, Xi’an, China) were placed by a trained neurophysiologist for FVEP and ERG (electroretinography) monitoring. The locations of the electrodes were determined according to the international 10–20 system [15]. The recording electrodes of the FVEP were situated at O1, O2, and OZ, while the reference electrode was located at FZ. To obtain a waveform with minimal interference, two ERG recording electrodes were positioned at the lateral canthi of both eyes, and the ground electrode was inserted into the deltoid muscle.

**Fig. 1:**
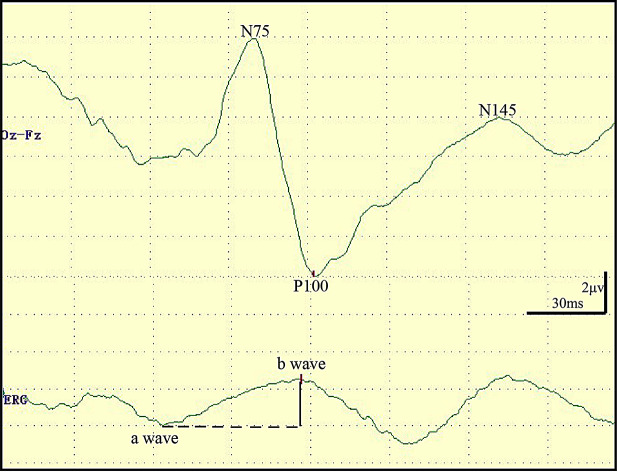
The typical waveform of FVEP and ERG. FVEP indicators were assessed by examining N75 to P100 amplitude and P100 latency. The ERG indicator was evaluated by examining a wave to b wave amplitude

A MEE-2000 neurophysiologic detection system (NIHON KOHDEN, Tokyo, Japan) was used for FVEP stimulation, recording, and measurement. During the whole operation, the patients wore a light emitting diode (LED) flash stimulation device (goggles, Unique Medical, Tokyo, Japan) with 20 red light that was covered with a transparent protective film to achieve real-time visual function monitoring. We set the monitoring parameters as follows: red light sourced of the stimulation mode, 1.0 Hz stimulation frequency, 1 to 100 Hz filter bandpass, and no less than 50 times the numbers of superpositions. Then, the FVEP followed by ERG was recorded at each time points approximately two or three times, and the typical waveform with less interference was used for data analysis. In our study, the vertical distance between N75 and P100 was defined as the VEP amplitude, the time to P100 evoked was defined as the VEP latency, and the vertical distance between a wave and b wave was defined as the ERG amplitude. The typical legends are shown in Fig. 1.

### Outcomes

The detection time points were set at T0 (approximately 20 min after intubation), T1 (approximately 20 min before the tumor resection), T2 (approximately 10 min after the tumor resection). The primary observation indicator was the N75-P100 amplitude of FVEP recorded at T0. The secondary observation indicators included the following: (i) the amplitudes at T1 and T2, the P100 latency at T0, T1 and T2, and the ERG b wave amplitudes at T0, T1 and T2. (ii) MAP, HR, BIS and PetCO_2_ at before anesthesia induction, T0, T1 and T2 for every patient in the two groups. (iii) the prevalence of postoperative dizziness, nausea and vomiting (PONV), the episodes of intraoperative awareness, and extubation time. (iiii) adverse events caused by subcutaneous needle electrodes, such as skin redness, periorbital bleeding or swelling, and peiorbital infection. Meanwhile, the changes in visual function, which were evaluated by comparing the pre- and postoperative corrected visual acuity using an international standard vision chart, where an improvement in visual acuity of 0.2 indicated improvement and a decrease of 0.2 indicated worsening.

### Sample size calculation

We used data from a recent study by PASS software 15.0 (NCSS, LLC. Kaysville, Utah, USA) for power analysis and sample size calculation. The mean (SD) of VEP N75-P100 amplitudes under remimazolam and propofol anesthesia were 7.69 ± 2.74 µV and 5.51 ± 2.07 µV, respectively [12]. We have established the non-inferiority margin (δ) for the FVEP amplitude under propofol anesthesia as the 10% level, with a value of 0.6. Using a one-sided, two-sample t test, 14 patients in each group can achieve 81% power to detect non-inferiority. The significance level (alpha) of the test is 0.025. Based on the assumed 20% dropout rate, 18 patients were required per group as a minimum sample size.

### Statistical analysis

Continuous variables are presented as the means ± standard deviation (`x ± s) or median (interquartile range [IQR]). The D’Agostino-Pearson omnibus normality test was used to evaluate the data distributions. The F test was used to compare variances between the two groups. Normally distributed data were analyzed with the unpaired t test or unpaired t test with Welch’s correction. Nonnormally distributed data were analyzed with the Mann-Whiteny test. Discrete variables were expressed as numbers and percentages (n [%]) and were analyzed with the fisher test or chi-square test.

The N75-P100 amplitude at T0 was tested for the non-inferiority of the group R to the group P. The 95% confidence interval (95% CI) showed the difference between the two groups. We used a significance level of α = 0.025 and a two-sided 95% confidence interval to test the non-inferiority hypothesis. We defined the 10% of the amplitude at T0 in the group P as the non-inferiority margin (δ). The efficiency of remimazolam was non-inferiority to that of propofol used for FVEP monitoring if the minimal value of CI was larger than − δ. SPSS 23.0 (IBM Corp, Armonk, NY, United States) and GraphPad Prism 8.0 (GraphPad Prism, Inc., San Diego, CA, USA) were used for data processing, data analysis, and diagram making. *P* < 0.05 was considered statistically significant.

## Results

### Demographic data

**Fig. 2:**
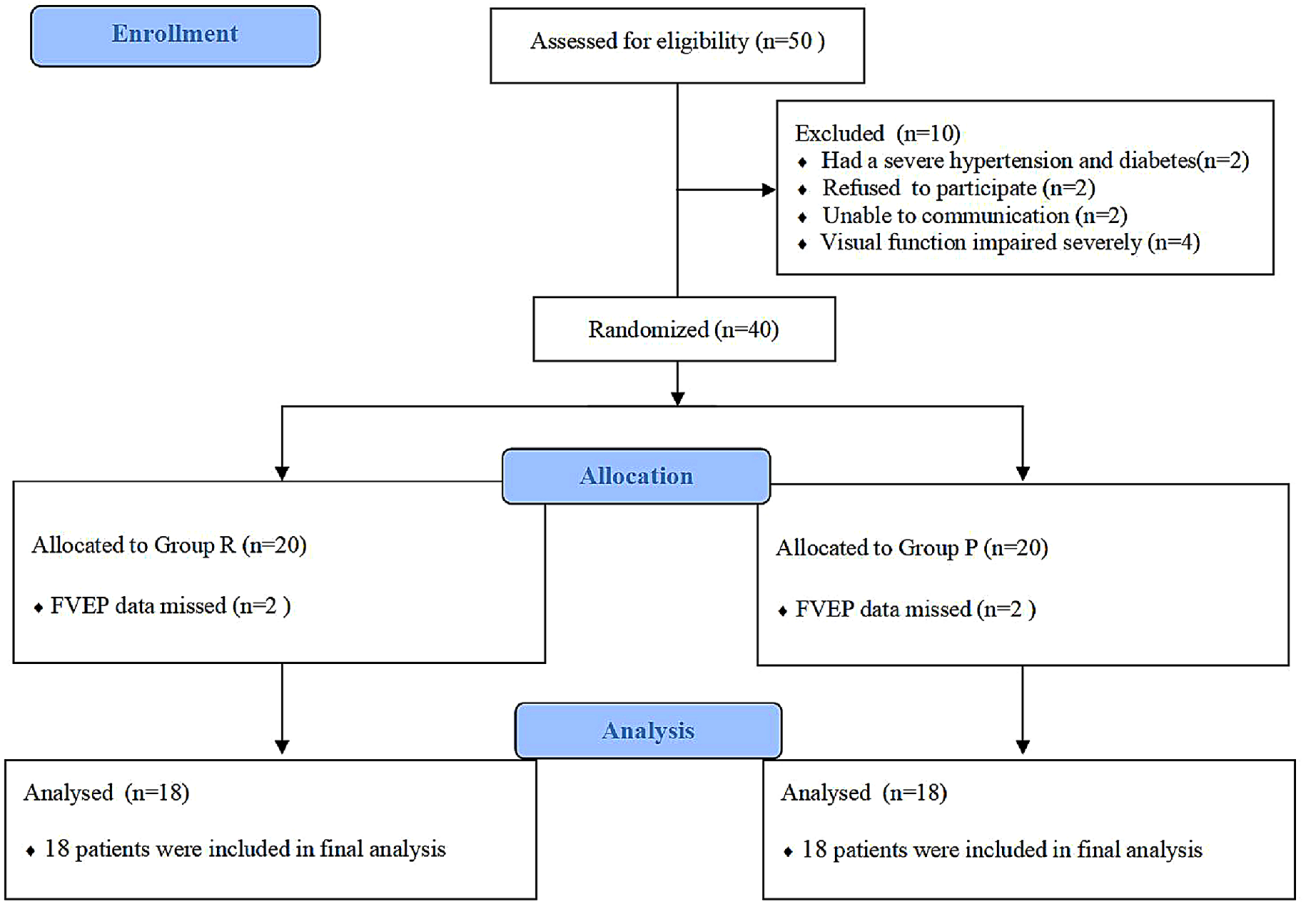
Flow diagram of patients

Initially, a total of 50 patients who underwent pituitary adenoma resection under general anesthesia were eligible for this study in our hospital. We excluded 10 patients from this study for the following reasons: severe hypertension or diabetes that was not controlled (n = 2); refusal to participate in the study (n = 2); inability to communicate with others well (n = 2); and severely impaired visual function and corrected visual acuity < 0.4 (n = 4). In the progress of data collection, 4 subjects were excluded due to FVEP data deficiencies. Finally, data for 36 patients were analyzed with 18 patients in each group (Fig. 2).

Table 1 shows the characteristics of the patients in the two groups. None of the patients in the two groups presented significant differences in terms of age, sex, height, weight, or body mass index (BMI) (*P* > 0.05). In group R, 10 patients presented headache, 6 patients appeared to have blurred vision, and 2 patients presented no typical symptoms. In group P, 6 patients exhibited blurred vision, 5 patients exhibited headache, 5 patients did not exhibit any typical symptoms, 1 patient exhibited irregular menstruation, and 1 patient exhibited acromegaly symptoms. For all patients in the two groups, the most common appearing symptom was headache (15 patients, 41.67%), followed by blurred vision (12 patients, 33.33%), none (7 patients, 19.44%) and others (2 patients, 5.56%). After tumor resection, the pathologist assessed the tumor diameters in the three dimensions using a, b, and c. The a, b, and c diameters of the tumor did not differ between the two groups (*P* > 0.05).

**Table 1:** Demographic characteristics between the two groups

Variables	Group R (n = 18)	Group P (n = 18)	*P*
Age (years)	58.94 ± 10.30	53.11 ± 14.97	0.182
Sex (male/female)	10/8	9/9	> 0.999
Height (cm)	166.30 ± 7.58	164.90 ± 6.98	0.587
weight (kg)	69.17 ± 11.74	72.28 ± 13.01	0.456
BMI (kg/m^2^)	24.86 ± 2.62	26.51 ± 4.36	0.179
Symptom n (%)			0.175
Blurred vision	6(33.33%)	6(33.33%)	
Headache	10(55.56%)	5(27.78%)	
None/physical examine	2(11.11%)	5(27.78%)	
Other	0(0.00%)	2(16.67%)	
Tumor diameter (cm)			
a	0.89 ± 0.27	1.04 ± 0.26	0.089
b	0.76 ± 0.32	0.94 ± 0.29	0.070
c	0.30 (0.20–0.50)	0.30 (0.30–0.40)	0.657

Variables presented as mean ± SD, median (interquartile range) or number of patients n (%). BMI, body mass index; the a, b and c diameters represented the tumor diameters in the three dimensions

The measured intraoperative variables are shown in Table 2. The duration of anesthesia, urine volume, blood loss, and transfusion volume did not show any significant differences between two groups (*P* > 0.05). Additionally, the PetCO_2_, HR and BIS index at each time point did not show any differences between groups (*P* > 0.05). However, the MAP at T1 in group P was lower than that in group R (*P* < 0.05). Overall, the blood pressure was maintained within the normal range.

**Table 2:** Intraoperative variables measurement

Variables	Group R (n = 18)	Group P (n = 18)	*P*
Duration of anesthesia (min)	187.1 ± 33.8	194.1 ± 39.5	0.568
Propofol dose (mg/kg/h)	—	5.3 ± 0.5	
Remimazolam dose (mg/kg/h)	0.8 ± 0.1	—	
MAP (mm Hg)			
Before the induction of anesthesia	104.5 ± 6.5	102.1 ± 6.6	0.272
T0	90.5 ± 7.9	89.2 ± 6.2	0.575
T1	83.5 ± 5.8	79.50 ± 5.4	0.039*
T2	85.9 ± 8.3	83.2 ± 8.7	0.346
HR (bpm)			
Before the induction of anesthesia	75.2 ± 13.2	76.6 ± 10.5	0.729
T0	67.1 ± 9.1	65.9 ± 5.9	0.668
T1	63.4 ± 6.8	65.2 ± 4.1	0.352
T2	62.5 ± 4.5	64.6 ± 4.4	0.164
BIS			
Before the induction of anesthesia	94.2 ± 2.6	94.8 ± 2.0	0.830
T0	48.6 ± 4.5	51.0 ± 3.9	0.098
T1	48.4 ± 3.9	47.8 ± 5.1	0.661
T2	49.8 ± 3.6	50.9 ± 5.2	0.484
PetCO_2_ (mm Hg)			
T0	32.8 ± 2.5	32.0 ± 2.3	0.303
T1	31.6 ± 2.6	31.4 ± 1.7	0.821
T2	32.3 ± 2.3	31.4 ± 1.6	0.186
Urine volume (ml)	600 (400–800)	600 (400–800)	0.542
Blood loss (ml)	50 (50–100)	50 (50-112.5)	0.164
Transfusion volume (ml)	1500 (1500–1775)	1500 (1375–2075)	0.418

Variables presented as mean ± SD, median (interquartile range). *P < 0.05 vs. Group P at the same time point. MAP, mean arterial pressure; HR, heart rate; bpm, beats per minute; BIS, bispectral index; PetCO_2_, pressure end-tidal CO_2_.

### FVEP monitoring data

**Fig. 3:**
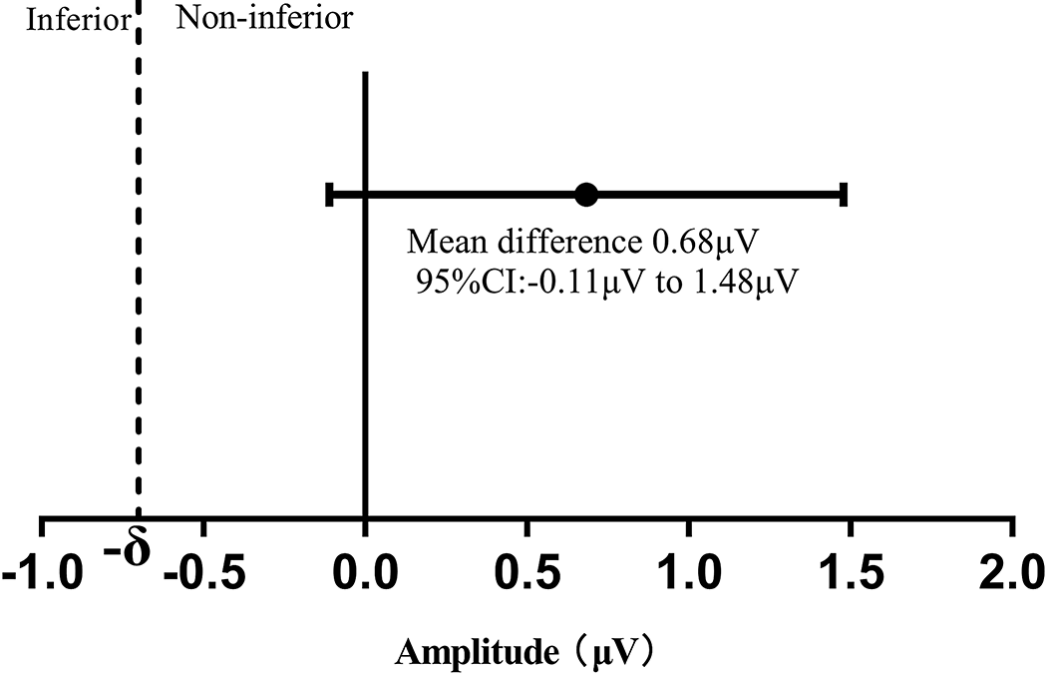
Mean difference in N75-P100 amplitudes at T0 between the two groups. Confidence interval testing was used for non-inferiority comparison. The right side of the dotted line indicates that group R was non-inferior to group P for N75-P100 amplitude. Error bars indicate 95% CI.

**Fig. 4:**
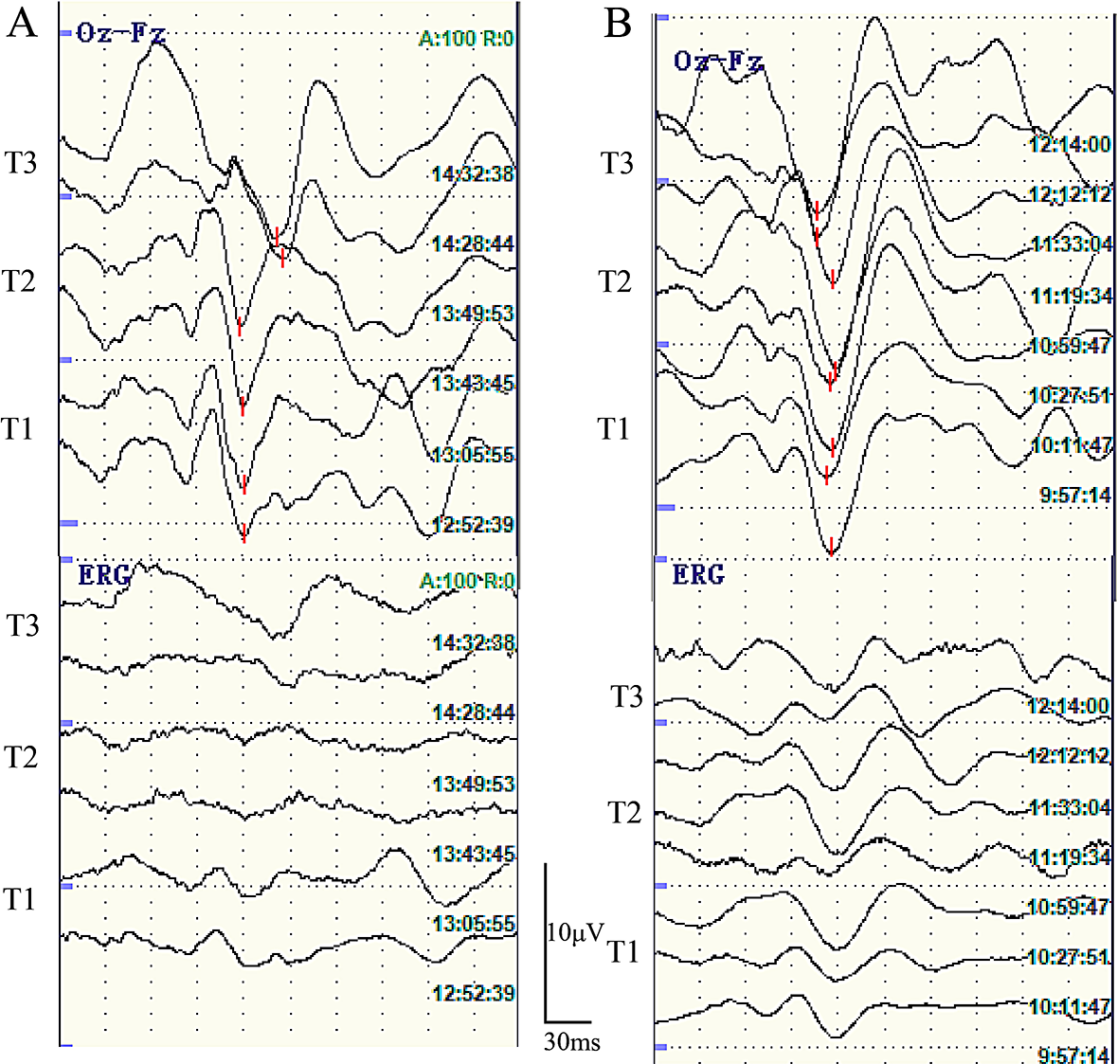
Typical waveforms of FVEP and ERG under remimazolam-0.6% sevoflurane general anesthesia (**A**) or propofol-0.6% sevoflurane general anesthesia (**B**). FVEP: flash visual evoked potentials, ERG: electroretinography

For the primary outcome in this study, the mean difference in the N75-P100 amplitude at T0 between both groups was 0.68 µV, with the 95% CI of -0.11µV to 1.48 µV (Table 3). The N75-P100 amplitude at T0 were 7.64 ± 1.36 µV in group R, 6.96 ± 0.95µV in group P (*P* = 0.09). The non-inferiority margin was set at 0.7 (10% amplitude at T0 in group P), and the lower limit of the 95% CI was greater than the -δ (Fig. 3). The typical waveforms of FVEP and ERG under general anesthesia in both groups during surgery are shown in Fig. 4. The elicitation rate of FVEP after general anesthesia in both groups was 100%, and reproducible and stable FVEP data were acquired at all time points. There were no significant differences between the two groups with regard to the FVEP amplitude at T1 and T2 or P100 latency at any time point (Table 3). In addition, we conducted an ERG wave analysis in conjunction with FVEP monitoring to confirm the successful delivery of flashing electrical stimulation to the retina. With regard to ERG b-wave amplitudes, there were no differences between the two groups (Table 3).

**Table 3:** Comparisons of FVEP and ERG b wave at T0, T1 and T2 between the two groups

Variables	Group R (n = 18)	Group P (n = 18)	Mean difference (95% CI)	*P*
N75-P100 (µV)				
T0	7.64 ± 1.36	6.96 ± 0.95	0.68 (-0.11 to 1.48)	0.09
T1	6.46 ± 1.41	6.18 ± 0.94	0.28 (-0.53 to 1.10)	0.483
T2	7.76 ± 1.26	7.28 ± 1.36	0.48 (-0.40 to 1.37)	0.275
P100 (ms)				
T0	111.70 ± 13.95	113.10 ± 12.56	-1.38 (-10.37 to 7.61)	0.756
T1	115.50 ± 13.89	117.70 ± 10.90	-2.28 (-10.74 to 6.18)	0.483
T2	119.10 ± 10.68	115.70 ± 8.79	3.44 (-3.18 to 10.07)	0.298
ERG b-wave (µV)				
T0	4.04 ± 1.40	3.70 ± 1.06	0.34 (-0.50 to 1.19)	0.411
T1	4.01 ± 1.28	3.59 ± 0.89	0.41 (-0.33 to 1.16)	0.270
T2	4.39 ± 1.56	4.07 ± 1.24	0.32 (-0.64 to 1.27)	0.504

Variables presented as mean ± SD. CI, confidence interval

### Adverse events

Postoperative adverse events related to general anesthesia, subcutaneous needle electrodes, and surgery were investigated and are shown in Table 4. The time to extubation in group R was not significantly different from that in group P (group R: 841 ± 73 s; group P: 870 ± 63 s, *P* > 0.05). The incidence of adverse events such as dizziness and PONV were not different between the groups (*P >* 0.05). No patients in either group experienced awareness during surgery. Subcutaneous needle electrodes did not cause severe adverse events, such as periorbital bleeding or swelling and periorbital infection. Postoperative corrected visual acuity worsening was not found in the two groups.

**Table 4:** Comparisons of adverse events related to anesthesia, electrodes and surgery post-operation between the two groups

Variables	Group R (n = 18)	Group P (n = 18)	*P*
extubation time (s)	841 ± 73	870 ± 63	0.102
dizziness n (%)	4(11.11%)	2(5.56%)	0.658
PONV n (%)	2(5.56%)	1(2.78%)	1.000
adverse events caused by electrodes n (%)			0.338
none	14(77.78%)	17(94.44%)	
skin redness	4(22.22%)	1(5.56%)	
periorbital bleeding or swelling	0(0.00%)	0(0.00%)	
periorbital infection	0(0.00%)	0(0.00%)	
postoperative visual acuity outcomes n (%)			1.000
unchanged	13 (72.22%)	14 (77.78%)	
improved	5 (27.78%)	4 (22.22%)	
worsened	0(0.00%)	0(0.00%)	

Variables presented as mean ± SD or number of patients n (%)

## Discussion

To date, there have been limited case reports detailing the impact of remimazolam on intraoperative evoked potential monitoring during neurosurgery [12, 13]. This prospective, randomized, controlled, and non-inferiority trial aimed to investigate whether stable and repeatable flash visual evoked potential waveforms can be obtained under remimazolam general anesthesia. Our findings indicate that the combination of remimazolam and 0.6% sevoflurane was comparably effective to the combination of propofol and 0.6% sevoflurane in terms of N75-P100 amplitude during pituitary adenoma resection. Furthermore, neither remimazolam nor propofol had a significant effect on the electroretinography b-wave amplitude. In addition, remimazolam combined with low concentrations of sevoflurane resulted in a satisfactory anesthesia environment with stable hemodynamics and moderate anesthesia depth, while limiting adverse events.

Previously, the flash VEP waveform was often considered unstable and difficult to reproduce, which limited its usefulness in neurosurgery. The clinical benefit of FVEP for monitoring visual pathway function has been debated due to factors such as the use of photostimulation devices and recording methods, as noted in previous studies [16–18]. However, in our study, we utilized high-intensity LED red flash goggles during surgery for real-time visual function monitoring. To ensure that the flash stimulation reached the retina, ERG monitoring was also recorded during surgery [19]. We simultaneously detected ERG following FVEP to avoid losing FVEP caused by retinal lesions or displacement of the flash stimulator. Additionally, subcutaneous needle electrodes were used in our study, which can acquire better signal intensity and resolution. Currently, epidemiology suggests that 30–70% of pituitary tumor patients will experience varying degrees of visual function defects [20]. In our study, 12 patients (33.33%) suffered from visual dysfunction, and experienced blurred vision before surgery. During pituitary tumor resection, it is possible to injure the optic nerve, making it more necessary for the surgeon to maintain reproducible and stable intraoperative FVEP to make a correct judgement. However, preoperative visual dysfunction may affect FVEP. A study by Kodama et al. demonstrated that stable VEP requires a preoperative visual acuity greater than 0.4 [21]. Another trial reported that a stable intraoperative VEP waveform can be acquired when visual acuity is ≥ 0.1 [5]. Therefore, our study only included patients with a visual acuity > 0.4 to avoid the influence of preoperative visual acuity on FVEP.

The physiological factors that affected the electrical signals of FVEP included hypotension, hypocapnia, hypoxemia, and deep anesthesia [7, 22, 23]. Table 2 shows the recorded intraoperative variables at each time point, including PetCO_2_, HR, BIS index, SpO_2_, and fluid balance, which were all maintained within the normal range. In addition, the MAP recorded at T2 in group R was higher than that in group P during anesthesia. This finding was consistent with a prior study [24] that the decrease in blood pressure was more moderate in the remimazolam group than in the propofol group during anesthesia. Overall, anesthesia with remimazolam or propofol combined with 0.6% sevoflurane can provide stable hemodynamics. Both groups failed to demonstrate persistent hypotension.

In addition to maintaining physiologic homeostasis, it is important to consider the effects of anesthetic drugs on FVEP amplitude and latency. Anesthetics can alter the transmission and activity of nerve cells and thereby impact the elicitation of electrical signals. Sevoflurane and propofol are both commonly used clinical general anesthetic drugs. Previous studies revealed that inhaled anesthetics can markedly inhibit VEP amplitude and latency, with the degree of effect positively correlated with concentration, while propofol slightly reduces VEP amplitude [6, 25, 26]. However, propofol has also been found to reduce FVEP amplitude, especially when administered in large doses [27]. Remimazolam, a new benzodiazepine drug, has been shown to enhance the function of γ-aminobutyric acid (GABA) receptors in the central nervous system, producing sedative and hypnotic effects [28]. A recent case series study also found that reproducible FVEP can be acquired under both remimazolam and propofol anesthesia [13]. Notably, Ma J et al. reported that a stable FVEP waveform can be elicited using a combination of propofol and 0.5 MAC sevoflurane [9]. Based on these studies, we compared the effects of remimazolam and propofol combined with 0.6% sevoflurane on the FVEP waveform. Our results showed that both groups had a 100% elicitation rate of FVEP, and reproducible and stable FVEP data were obtained. There were no significant differences between the two groups in terms of FVEP amplitude and latency at the T0, T1 and T2 time points.

The FVEP, a signal formed in the visual cortex when the eyes receive a flash-induced stimulus, has a highly variable waveform due to the effects of anesthesia, particularly sensitivity to inhaled anesthetics. When sevoflurane and propofol are utilized at clinical doses, they may demonstrate discrepant effects on the FVEP. The distinct molecular targets and neuronal pathways [29] can give rise to variations in their respective impacts on the FVEP. Volatile anesthetic agents are known to reduce the excitability of neurons mediated by N-methyl-d-aspartic acid (NMDA) and GABA receptors, while propofol and remimazolam primarily enhance neural suppression mediated by GABA receptors [30–32]. Consequently, this may explain the varying effects of anesthetics on FVEP.

During surgery, it is important to minimize body movement responses when using remimazolam anesthesia [32], as well as to reduce the inhibitory effect of sevoflurane on FVEP. Although previous studies have verified the depth of remimazolam-based total intravenous anesthesia for patients, prolonged high-dose remimazolam infusion would lead to a delay recovery of consciousness [33, 34]. Taking both drug aftereffects and FVEP signals into account, we applied a 0.6% sevoflurane combined with remimazolam anesthesia protocol. Not coincidentally, a study of rats revealed that low concentrations of sevoflurane also presented satisfactorily repeatable FVEP signals over time [35]. Additionally, Ma J demonstrated that 0.5 MAC or less of sevoflurane is suitable as an optional anesthesia regimen for FVEP monitoring [9]. Overall, the anesthetic states observed in our study, no patients experienced delayed recovery and reproducible FVEP was successfully elicited at the designated time points under remimazolam-0.6% sevoflurane anesthesia.

Our study has some limitations. First, the FVEP data of patients before anesthesia were not obtained in our study. It was difficult for patients to tolerate the pain during subcutaneous needle electrode insertion. Second, the trial was a single center study, and the included sample size was small. Multicenter studies are required to further verify the conclusions of our study. Third, we only analyzed FVEP data from both eyes. In further studies, FVEP data from each eye should be collected in a timely manner. Fourth, we adopted a balanced protocol utilizing less than 0.5 MAC sevoflurane after comprehensive assessments of remimazolam aftereffects and FVEP acquiring. The use of 0.6% sevoflurane may obtund the difference between remimazolam and propofol.

## Conclusions

We concluded that remimazolam-sevoflurane was not inferior to propofol- sevoflurane in terms of FVEP N75-P100 amplitude for pituitary adenoma resection. Additionally, both remimazolam and propofol in conjunction with 0.6% sevoflurane had little impact on the ERG-b wave. The reproducibility and stability of the FVEP and ERG waveforms were consistent across both groups during anesthesia. Therefore, the utilization of remimazolam combined with 0.6% sevoflurane appears to be a safe and effective strategy for ensuring reliable FVEP monitoring.

## Data Availability

All authors agreed to share the datasets related to this study after the article was published. The original data in our study can be shared with readers from the corresponding or the first authors on reasonable request.
